# A Relational Adaptive Neural Model for Joint Entity and Relation Extraction

**DOI:** 10.3389/fnbot.2021.635492

**Published:** 2021-03-16

**Authors:** Guiduo Duan, Jiayu Miao, Tianxi Huang, Wenlong Luo, Dekun Hu

**Affiliations:** ^1^School of Computer Science and Engineering, University of Electronic Science and Technology of China, Chengdu, China; ^2^Trusted Cloud Computing and Big Data Key Laboratory of Sichuan Province, Chengdu, China; ^3^Department of Fundamental Courses, Chengdu Textile College, Chengdu, China; ^4^College of Computer, Chengdu University, Chengdu, China

**Keywords:** entity relation joint extraction, overlapping triplets detection, DCGCN, relational-adaptive mechanism, graph convolutional networks

## Abstract

Relation extraction is a popular subtask in natural language processing (NLP). In the task of entity relation joint extraction, overlapping entities and multi-type relation extraction in overlapping triplets remain a challenging problem. The classification of relations by sharing the same probability space will ignore the correlation information among multiple relations. A relational-adaptive entity relation joint extraction model based on multi-head self-attention and densely connected graph convolution network (which is called MA-DCGCN) is proposed in the paper. In the model, the multi-head attention mechanism is specifically used to assign weights to multiple relation types among entities so as to ensure that the probability space of multiple relation is not mutually exclusive. This mechanism also predicts the strength of the relationship between various relationship types and entity pairs flexibly. The structure information of deeper level in the text graph is extracted by the densely connected graph convolution network, and the interaction information of entity relation is captured. To demonstrate the superior performance of our model, we conducted a variety of experiments on two widely used public datasets, NYT and WebNLG. Extensive results show that our model achieves state-of-the-art performance. Especially, the detection effect of overlapping triplets is significantly improved compared with the several existing mainstream methods.

## Introduction

How to extract semantic and structured data from unstructured text is a particularly important task in the era of big data. Entity relation extraction is an essential subtask in the field of natural language processing (NLP). Its goal is to identify entity pairs from the text and extract one or more semantic relations between entity pairs, as shown in Table 1. The extracted triples are used extensively in many downstream NLP tasks, such as knowledge graph construction (Luan et al., 2018), intelligent question answering system (Yang et al., 2019).

**Table 1:** An example of an overlapping triplet.

**The [United States] president [Donald Trump] was born in [New York City]**.	
SingleEntityOverlap **(SEO)**	EntityPairOverlap **(EPO)**
(**Donald Trump**, President_ of, United States)	(**Donald Trump**, Governance, **UnitedStates**)
(**Donald Trump**, Born_ in, New York City)	(**Donald Trump**, President Of, **United States)**

At present, the relation extraction method can be divided into pipeline method and joint extraction method according to entity recognition and relation extraction, whether two subtasks are completed in order at one time. The traditional method was to adopt pipeline model, in which entity recognition was carried out first and then entity pair relation extraction was carried out. Two extraction models were used respectively. This method has high flexibility and does not need to annotate the dataset of entities and relations at the same time. However, this method makes the model have error accumulation problem and the interaction information is missing (Li and Ji, 2014), ignoring the internal correlation information between the two tasks. Therefore, more work now focuses on the method of joint learning and makes the most of interactive information between entities and relations, which can solve the above problems to a certain extent. Some joint learning methods treat relation extraction as a sequential tagging problem, which cannot solve the words with multiple tags and therefore cannot extract relation triples with overlapping entities. As shown in Table 1, there may be different relations between the same entity pair in some sentences, such triples are called EntityPairOverlap (EPO), or there is one same entity between entity pairs, and such triples of relation are called SingleEntityOverlap (SEO). The extraction of overlapping triples is particularly difficult for the relational extraction model of joint learning, because there are no entities in the input and the entities need to be recognized by the model. In practical application, there are a large number of overlapping triples in text as shown in Table 1, and such text data will bring troubles to the current sequence tags-based joint learning methods. Therefore, effectively solving the problem of overlapping triples extraction can greatly improve the performance of the joint learning model.

Therefore, relation extraction still faces the great challenge of triplet extraction of overlapping entities and extraction of multiple relations between entity pairs. When detecting multiple relation types between entity pairs, most existing studies (Zeng et al., 2018; Nayak and Ng, 2020) are usually regarded as a multi-classification task, in which multiple relationship types share the same probability space. In the final classification, multiple relationships will be mutually exclusive, so the use of classifier detection will reduce the correlation degree of relationships. When detecting overlapping triples, the dependency information of words and the interaction information of triples are also significant. The method ImprovingGCN (Hong et al., 2020) and AntNRE (Sun et al., 2019) consider the dependency information of words and the interaction of triplets, but they do not consider the interaction information between the probabilistic subspaces of different relation types. Thus these methods ignore the high correlation between multiple relations and entity pairs.

In order to address the above issues, we propose a relational-adaptive joint entity relation extraction model based on multi-head self-attention and densely connected graph convolutional networks (DCGCN). Firstly, the model extracts the multi-granularity feature information from the text through the feature mixed encoding layer, so that the subsequent model can better capture the semantic information of the sentence. Then we get further dependency information between words through the stacked LSTM and GCN. In addition, we use the multi-head self-attention mechanism to assign weights to multiple relation types among entities so as to ensure that the probability space of multiple relations is not mutually exclusive, and extract the interaction information between the relation and entity. This method can construct multiple dynamic association matrices for each sentence, which can be used as the input of the second phase DCGCN to consider the interaction information between the probabilistic subspaces of different relation types. The DCGCN carries on the interaction of entity and relation in the second phase to obtain the structural information and potential text semantic information of the deeper level graph. Finally, entities and relations are predicted through the node representations extracted from the two-phase GCN.

The contributions of our work are summarized as follows:

(1) We propose a new joint entity relational extraction method based on two-phase GCN, which is an end-to-end model. The GCN in the first phase obtains the dependency information between words by inputting multi-granularity semantic features, while the DCGCN in the second phase can capture the potential semantic association between words in a specific relationship by inputting multiple attention dynamic association matrices.
(2) We design a relation adaptive mechanism based on multi-head attention to learn different relation types between overlapping entity pairs. This mechanism allocates different attention weights to the relations between entity pairs, and adaptively identifies the relations between entity pairs. This method can effectively identify overlapping triples.
(3) Extensive experiments have been conducted with the method in this paper, and the results indicate that our model achieves state-of-the-art performance on two widely used public datasets.

The following of paper is structured as follows. In Section, Related Works are provided, followed by a detailed description of the proposed model MA-DCGCN in Section Methodology. Our proposed framework is evaluated on two public datasets in Section Experimental and Results. The conclusion is drawn in Section Conclusion.

## Related Work

The traditional pipeline method, in which the model is mainly based on the existing CNN (Zeng et al., 2014; Zhu et al., 2017), RNN (Socher et al., 2012; Hashimoto et al., 2013), LSTM (Xu et al., 2015; Zhang et al., 2015), ameliorates the performance of the model by changing the input characteristics or network architecture of the model. Due to its natural advantages in processing unstructured data, GCN has gained increasing popularity, which was introduced in many works to learn the rich information contained in the dependency tree (Zhang et al., 2018, 2019; Guo et al., 2019). Qian et al. (2019) have improved word-level information extraction by constructing a complex graph structure with multiple relationships, and then using GCN to propagate information between nodes to generate rich features.

The initial linkage between entity recognition and relation extraction is established in the NovelTagging model (Zheng et al., 2017), which unifies the two tasks as a single sequential tagging problem. However, this method cannot resolve words with multiple tags, and therefore cannot extract relational triples with overlapping entities. Miwa and Bansal (2016) proposed a model based on bidirectional LSTM-RNN to represent the parameters of entity recognition and relation extraction jointly, but its model learning process is still similar to pipeline method, and it is not a typical joint extraction method. Katiyar and Cardie (2017) proposed for the first time a real entity relation joint extraction model based on recursive neural network, which does not rely on any dependency tree information, models entity recognition sub-tasks into sequence annotation tasks, and then extracts the relation between entity pairs through Shared coding layer features. Reinforcement learning (RL) is also widely used in the field of relation extraction (Qin et al., 2018; Takanobu et al., 2019; Zeng et al., 2019), in which the remote supervised noisy data sets are used to jointly optimize entity recognition module and relation extraction module.

For the sake of overcoming the problem of the redundant entity in the previous methods, Zheng et al. (2017) proposed an end-to-end sequence tag, the relation between extraction task as a tagging task. Sun et al. (2019) proposed binary entity relation graph to run on a new type of graph convolution network (GCN) after the graph on the binary map convolution computation, and the model can capture the entities and relations between the effective information. Chen et al. (2020) proposed a multi-channel framework composed of layered deep neural networks stacked to achieve relation extraction at sentence level. The above works can simultaneously extract entities and relations through joint extraction, but the model has poor performance for triples of overlapping relations.

Zeng et al. (2018) proposed a neural model CopyRE based on Seq2Seq for the first time in view of the overlapping relation, and the model considered the overlapping problem of relational triad through the copying mechanism of the entity copied from the source statement by the decoder. Fu et al. (2019) proposed the GraphRel model of end-to-end relation extraction for entity overlap. The model was divided into two phase, and the interaction between entities aand relations was considered through the relational weighted GCN of the second phase, which significantly improved the prediction of overlapping relations. Yuan et al. (2020) proposed a joint entity and relationship extraction model called RSAN, which combined the fine-grained semantic information of the relation to guide the entity recognition process. Zeng et al. (2020) proposed their own improved model CopyMTL and introduced named entity task for multi-task learning based on CopyRE, thus improving the problem that CopyRE can only extract single words and cannot match multi-character entities. Hong et al. (2020), based on GraphRel, proposed a new relational perceptive attention mechanism, which can acquire the representation of the relation between the span of two entities. This model utilized the characteristics of adjacent nodes and edge information when obtaining the characteristics of the encoding node. To solve the overlapping triple problem, we use the stacked LSTM-GCN encoder to identify entities, and introduce the multi-head self-attention mechanism to identify the relation types of overlapping entity pairs according to different attention weights, and then use the densely connected graph GCN to further extract the interaction information between entities and relations. The model implements end-to-end entity recognition and relation extraction through joint training of loss functions in different phases.

## Methodology

**Figure 1:**
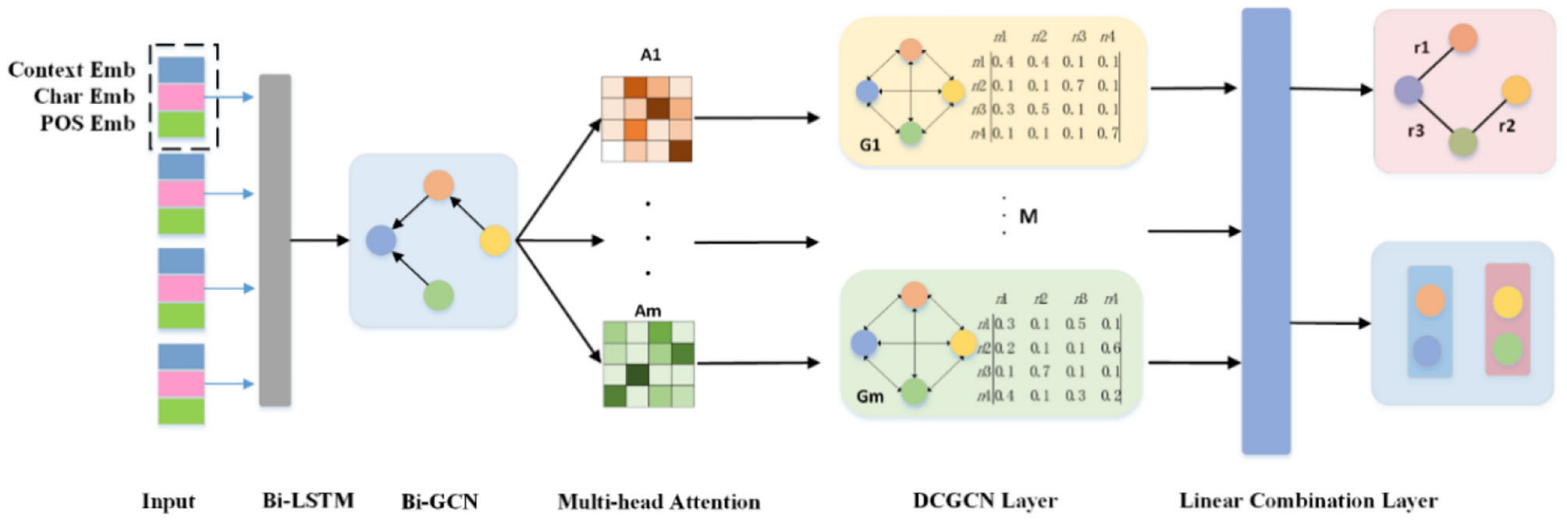
The MA-DCGCN model for joint entity and relation extraction.

In this section, we introduce a relational extraction model for relational-adaptive densely connected graph convolutional network model using a multi-head self-attention mechanism which called MA-DCGCN. This model can extract the triples of overlapping relations in an end-to-end method. As shown in Figure 1, our model consists of four parts: the LSTM-GCN encoding layer, relation-adaptive multi-head attention layer, dense connected DCN layer and the linear combination layer.

### Encoding Layer

Given a text sequence *S* = {*w*_1_, *w*_2_, ⋯ , *w*_*i*_, ⋯ , *w*_*n*_} of length *n*, where *w*_*i*_ represents the *i*-th word in the sentence. First, we represent the text sequence as feature matrix *X* = [*x*_1_, ⋯ , *x*_*i*_, ⋯ , *x*_*n*_], and the i-th input word is initially represented as *x*_*i*_. *x*_*i*_ is composed of word context embedding, part-of-speech (POS) embedding and character-based word features. By inputting text sequences into a pre-trained Bert model to obtain context embedding, the model can be provided with the contextual semantic characteristics of word sequences. Character-based word features are computed by a convolutional neural network on a text sequence (CNN) (Krizhevsky et al., 2017).

(1) $$\begin{array}{r} x_{i}=Context\left(w_{i}\right)⊕POS\left(w_{i}\right)⊕Char\left(w_{i}\right) \end{array}$$

Recursive neural network (RNN), long short-term memory network (LSTM) (Schuster and Paliwal, 1997) and gated recursive unit (GRU) are all effective methods for long sequence modeling (Hochreiter and Schmidhuber, 1997; Cho et al., 2014). For the sake of full consideration of the context semantic information of text sequence and the dependencies between words, we adopt bidirectional LSTM (Bi-LSTM) to encode the input word *x*_*i*_ and its context together. The forward and backward LSTM hidden states are concatenated to obtain the complete context-aware vector *h*_*i*_ of the word *x*_*i*_ in the time step i. The expression formula of *h*_*i*_ is as follows:

(2) $$\begin{array}{r} {\vec{h}}_{i}=LSTM_{F}\left(x_{i},{\vec{h}}_{i-1}\right), \end{array}$$

(3) $$\begin{array}{r} {\overset{\leftarrow }{h}}_{i}=LSTM_{B}\left(x_{i},{\overset{\leftarrow }{h}}_{i-1}\right), \end{array}$$

(4) $$\begin{array}{r} h_{i}=\left[{\vec{h}}_{i};{\overset{\leftarrow }{h}}_{i}\right],i\in \left[1,n\right] \end{array}$$

Where, $h_{i}\in R^{2\times d_{l}}$, *d*_*l*_ stands for the dimension of Bi-LSTM's hidden state, *F* and *B* stand for the two directions of forward and backward of LSTM respectively. $h_{i}^{0}$ is the initial input feature *x*_*i*_, $x_{i}\in R^{d}$, and *d* is the dimension of the input feature.

In our work, the representation of the calculated output of the Bi-LSTM encoder serves as the input to the next Bi-GCN. For a given graph with *n* nodes, the nodes in the graph are each word in the sentence, and the edges in the graph are the dependencies between words. We use *n* × *n* adjacency matrix *A*_*ij*_ to represent the graph, and we add a self-loop for each point, that is, *A*_*ij*_ = 1(*i* = *j*). When there is a dependency relation between word *i* and word*j*, *A*_*ij*_ = *A*_*ji*_ = 1, otherwise it is 0. Given the representation of layer *l*, we can derive the representation of layer *l*+1 from the following formula.

(5) $$\begin{array}{r} {\vec{h}}_{i}^{\left(l+\text{1}\right)}=\rho \left(\underset{j\in \vec{N}\left(i\right)}{\sum }A_{ij}{\vec{W}}^{\left(l\right)}h_{j}^{\left(l\right)}+{\vec{b}}^{\left(l\right)}\right) \end{array}$$

(6) $$\begin{array}{r} {\overset{\leftarrow }{h_{i}}}^{\left(l+1\right)}=\rho \left(\underset{j\in \vec{N}\left(i\right)}{\sum }A_{ij}{\overset{\leftarrow }{W}}^{\left(l\right)}h_{j}^{\left(l\right)}+{\overset{\leftarrow }{b}}^{\left(l\right)}\right) \end{array}$$

(7) $$\begin{array}{r} {\vec{h}}_{i}^{\left(l+\text{1}\right)}=\left[{\vec{h}}_{i}^{\left(l\right)};{\overset{\leftarrow }{h_{i}}}^{\left(l\right)}\right],i\in \left[1,n\right] \end{array}$$

Where, *W* and *b* are the weight matrix and deviation, *Ni* is the neighbor of node *i*, and ρ is the activation function (such as *RELU*, etc.).

By extracting the word features from LSTM-GCN encoding layer, we can recognize the entity of the word and predict the relation between word pairs. For entity recognition, we apply classification loss to the word features obtained by LSTM, denoting as *L*_*ner*1_.

(8) $$\begin{array}{r} P\left(ŷ|w_{i},s\right)=soft\text{ max}\left(W_{ner\text{1}}h_{i}+b_{ner\text{1}}\right) \end{array}$$

(9) $$\begin{array}{r} L_{ner1}=\;-\frac{1}{m}\sum_{i=1}^{m}\log P\left(ŷ=y|w_{i},s\right) \end{array}$$

Regarding the relation extraction, we learn the weight matrix $W_{r}^{i},W_{r}^{j}$ for the relation *r* of word pairs (*w*_*i*_, *w*_*j*_), and calculate the fraction *S* of word pairs (*w*_*i*_, *w*_*j*_) under the relation *r*. By calculating the probability of each relation between word pairs, we can get the relation of this phase to extract loss *L*_*re*1_.

(10) $$\begin{array}{r} S_{r}\left(w_{i},w_{j}\right)=RELU\left(W_{r}^{i}h_{w_{i}}⊕W_{r}^{j}h_{w_{j}}\right) \end{array}$$

(11) $$\begin{array}{r} P_{r}\left(w_{i},w_{j}\right)=soft\text{ max}\left(S_{r}\left(w_{i},w_{j}\right)\right) \end{array}$$

(12) $$\begin{array}{r} L_{\text{re}1}=-\frac{\text{1}}{\text{m}}\overset{m}{\underset{i=1}{\sum }}\log P_{r}\left(w_{i},w_{j}\right) \end{array}$$

### Relation-Adaption Multi-Head Attention Layer

To work out the difficult overlapping problem of relations, we applied DCGCN again on the graph after the LSTM-GCN encoder layer, further propagated and learned the information of entities and relations on the constructed word graph. Considering that the edge information of the graph also contains information that is beneficial to entity relation extraction, the multi-head self-attention mechanism is added instead of using DCGCN directly in the second time, which can allocate an exclusive probability subspace for each relation between entity pairs without mutual exclusion. Based on the relation-adaption mechanism, we can calculate the independent correlation strength for the entities under different relation types in the sentence according to the semantic characteristics of the context, and detect the relation types between entity pairs adaptively. The attention matrix *A*^*m*^ ∈ *R*^*N* × *N*^ calculated by us is as follows:

(13) $$\begin{array}{r} A^{\left(m\right)}=soft\text{ max}\left(\frac{Q^{m}\overset{m}{\underset{Q}{W}}\times {\left(K^{m}W_{K}^{m}\right)}^{T}}{\sqrt{d_{r}}}\right)V^{m} \end{array}$$

Where $Q^{m}\in R^{N\times d_{r}}$, $K^{m}\in R^{N\times d_{r}}$ represents the query and key of the *m*-th relational type, matrix *W* is the model parameter, and *d*_*r*_ is the dimension of the subspace of each relational type. $A_{i,j}^{\left(m\right)}$ represents the strength of the association between the word *i* and the word *j*in the *m*-th relation.

### Densely Connected GCN

In our work, a shallow GCN captures only local structural information on a large graph built of all words based on text sequences. Inspired by DenseNet (Huang et al., 2017) in the field of neural networks, we introduce densely connected GCN (Guo et al., 2019) into our MA-DCGCN model in order to capture richer node-related non-local information on large graphs for entity relation learning.

**Figure 2:**
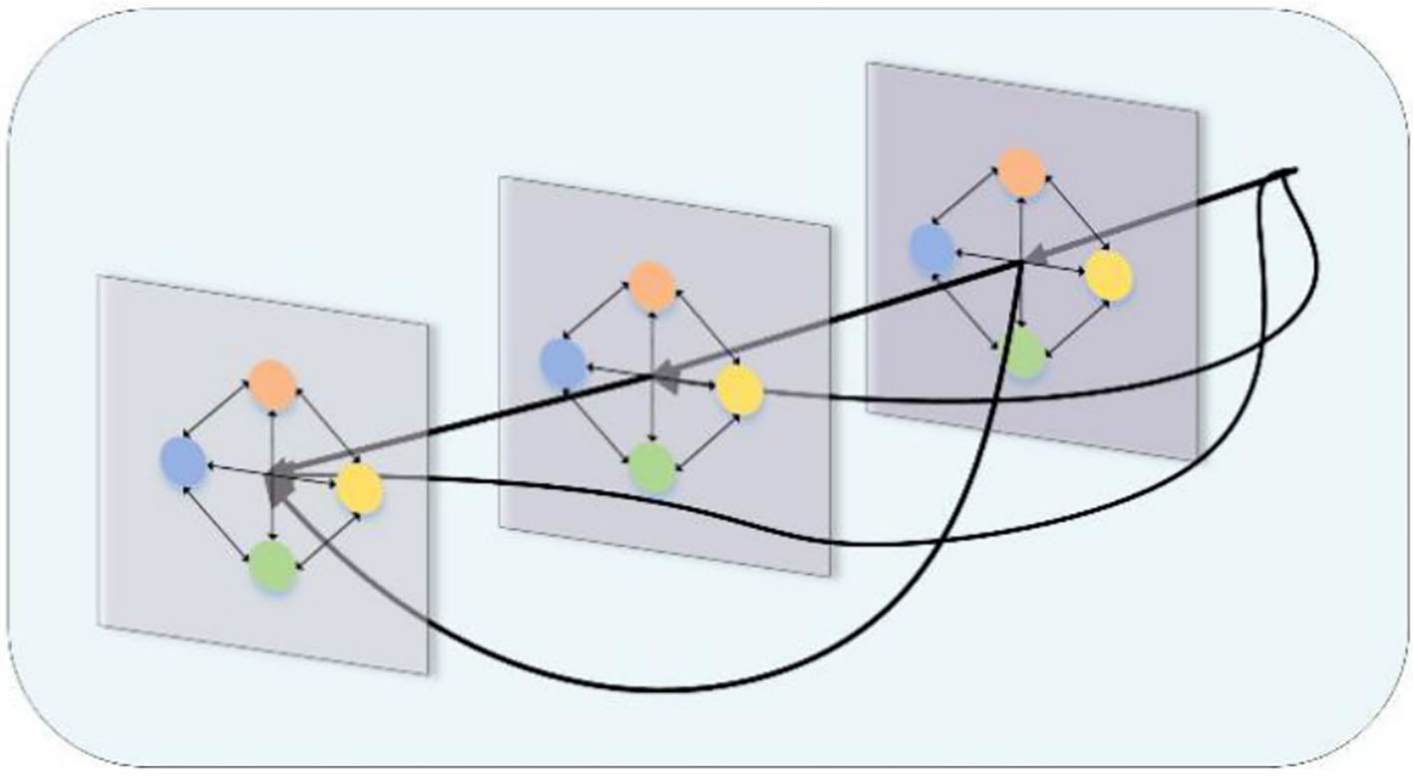
An illustration of a three-layer densely connected graph convolutional network.

The structure of a densely connected GCN at three layers is shown in Figure 2, with any layer receiving the output of all preceding layers. For example, outputs from the first and second layers can be input to the third layer, so that first-order, second-order, and third-order neighborhood information from the nodes can be received. By using dense connections, we can train deeper GCN models to produce richer graphical representations than shallow GCN.

In densely connected GCN, the features of node *v* in Layer *l* not only contain the output feature *h*^(*l*−1)^ of layer *l*−1, but also input the feature information of all previous layers. The node features $g_{v}^{\left(l\right)}$ of layer *l* are denoted by the series of initial node feature *x*_*i*_ and nodes of all previous layers:

(14) $$g_{i}^{\left(l\right)}=[x_{i};\;h_{i}^{\left(1\right)};\;\ldots ;\;h_{i}^{\left(l-1\right)})]$$

Since we generate independent subspaces for M relation types that are not mutually exclusive, we need to run m densely connected GCN layers for M attention matrices, so the GCN calculation is modified as follows:

(15) $$\begin{array}{r} h_{m_{i}}^{\left(l\right)}=RELU\left(\overset{m}{\underset{j\in N\left(i\right)}{\sum }}A_{ij}^{\left(m\right)}W_{m}^{\left(l\right)}g_{j}^{\left(l\right)}+b_{m}^{\left(l\right)}\right) \end{array}$$

Where, *m* = 1, 2, ..., *M*, *W* and b are the parameter matrix and bias terms associated with the attention matrix A. Each layer of W's dimension increases *d*_*hidden*_, which is determined by the number of densely connected layers *L* and the input feature dimension d. In this paper, *L* = 3. And $w^{\left(l\right)}\in R^{d_{hidden}\times d^{\left(l\right)}}$, where $d^{\left(l\right)}=d+d_{hidden}\times \left(l-1\right)$.

In order to integrate the feature representation of M relational types that have been closely connected, we use a general linear combination layer to output the final word features.

(16) $$\begin{array}{r} h_{final}=W_{final}\left[h_{1}^{};...;h_{M}^{}\right]+b_{final} \end{array}$$

Where $W_{final}\in R^{d\times M}$ is the weight matrix, and *b* is the bias vector of the linear transformation. Using the word features we finally obtained, we performed entity recognition and relation classification in Section “Encoding Layer” again to obtain that the losses in this phase were represented as *L*_*ner*2_ and *L*_*ner*2_.

### Joint Extraction

We adopt two kinds of losses in our joint training, entity recognition loss and relation extraction loss. For entity recognition, we use common BIESO marking scheme to represent the real labels, every word for text sequence must belong to one class. The total loss of our joint training is equal to the sum of two entity recognition losses and two relationship extraction losses in the whole calculation process of the model. The calculation formula of the total loss is as follows:

(17) $$\begin{array}{r} L=\left(L_{ner1}+L_{re1}\right)+\alpha \left(L_{ner2}+L_{re2}\right) \end{array}$$

Where α is the weight between the losses of the two phases. Our model is trained jointly by minimizing L.

## Experimental and Results

### Dataset

We evaluate our model's performance on two public datasets that are common in the field of relational extraction.

**New York Times (NYT)**: the New York Times data set contains the New York Times web site from November 2009 to January 2010 in the 150 articles on business. The New York Times data set is constructed using a remote monitoring method, which generates large-scale training data by automatically aligning relations in Freebase with text content. NYT contains 24 valid relations. This paper's work refers to (Zheng et al., 2017) to preprocess the original NYT data set.

**WebNLG**: WebNLG was originally a data set for natural language generation (NLG) by Gardent et al. (2017), containing 246 valid relations. In this dataset, an instance consists of a set of triples and a few standard sentences (written by the annotator). This paper only uses the first sentence in each instance in WebNLG dataset, which needs to contain all the entities of the triples, otherwise the sentence is filtered.

The final NYT and WebNLG statistics for the three types of triples are shown in the following Table 2.

**Table 2:** Statistics about the datasets.

**Category**	**NYT**		**WebNLG**	
	**Train**	**Test**	**Train**	**Test**
Normal	37,013	3,266	1,596	246
EPO	9,782	978	227	26
SEO	1,4735	1,297	3,406	457
All	5,6195	5,000	5,019	703
Relation	24		246	

### Implementation Details

PyTorch has been used in our work to distribute GCN and DCGCN in node neighborhood information and edge feature information. We used pre-trained BERT to initialize the context embedding of the word (768d) and then concatenate it with trainable POS embedding (15d) and character-level features (25d) as the input for each word. The dimension of the hidden state vector for Bi-LSTM is set to 100, and the dimension of the hidden state vector for Bi-GCN, attention layer, and DCGCN is set to 256. We selected 10% randomly from the training set to optimize the super parameters in the model, the Learning Rate, Dropout and batch size were set to 0.0001, 0.1, and 10, respectively, and Adam optimizer was used in the model.

In our model, we set the number of layers of BI-GCN to be 2, and the number of layers of densely connected GCN is *L* = 3. Parameter α is set to 3 for joint training.

### Baselines and Comparison Result

To verify the superior performance of the model in this paper, we compare it with a series of recent related models and we contrast it with some mainstream models listed below.

- NovelTagging (Zheng et al., 2017) proposes an end-to-end model based on LSTM and adopts a new tagging method to solve the task of joint extraction of entities and relations.
- GraphRel (Fu et al., 2019) extracts the hidden features of nodes through the stacked GCN of two stages, and trains the loss function of two stages together to realize the joint extraction of entities and relations.
- AntNRE (Sun et al., 2019) detects the entity span by sequence tagging, deduces the entity relation type based on GCN in the entity-relation bigraph, and trains the two subtasks jointly.
- CopyRe (Zeng et al., 2018) uses two different decoding strategies to generate relations, and then extracts entities and relations jointly based on the copy mechanism. We compare the results with the MultiDecoder.
- CopyMTL (Zeng et al., 2020) introduces a multi-task learning framework, which solves the problem of extracting only one word in CopeRe by adopting different strategies for the head entities, tail entities and relations in triples.
- OrderRL (Zeng et al., 2019) regards the extraction of triples as a process of reinforcement learning (RL), explores the influence of the extraction order of triples, and the proposed sequence-to-sequence model can automatically learn and generate relational facts.
- HRL (Takanobu et al., 2019) applies reinforcement learning to a new joint extraction paradigm, and the proposed hierarchical reinforcement learning (HRL) model decomposed the entity and relation extraction process into a two-level RL strategy hierarchy.
- ImprovingGCN (Hong et al., 2020) improves on the basis of GraphRel and added the attention mechanism, allowing the model to use the weighted edge information on the graph structure. The proposed model can be used to end-to-end extract entities and relations jointly.

In this paper, three indexes, precision, Recall and F1, which are the same as most relationship extraction work, are used to assess the performance of the model. The comparison results are shown in the Table 3 below.

**Table 3:** Results of comparison with mainstream methods on NYT and WebNLG datasets.

	**NYT**			**WebNLG**		
	**Precision**	**Recall**	**F1**	**Precision**	**Recall**	**F1**
	**(%)**	**(%)**	**(%)**	**(%)**	**(%)**	**(%)**
NovelTagging	62.4	31.7	42.0	52.5	19.3	28.3
CopyRe	61.0	56.6	58.7	37.7	36.4	37.1
GraphRel	63.9	60.0	61.9	44.7	41.1	42.9
CopyMTL	75.7	68.7	72.0	58.0	54.9	56.4
OrderRL	77.9	67.2	72.1	66.3	59.9	61.6
HRL	78.1	77.1	77.6	-	-	28.6
AntNRE	80.2	53.5	64.2	80.4	45.4	58.0
ImprovingGCN	83.2	64.7	72.8	66.4	62.7	64.5
Ours	81.3	76.7	**79.4**	67.4	**65.1%**	**66.3%**

*Bold marks highest number among all models*.

As shown in Table 3, we compared our work with the above baseline model, which can verify the effectiveness of our model MA-DCGCN. Similar to GraphRel (Fu et al., 2019) and improving GCN (Hong et al., 2020), our work uses GCN. But the difference is that our model applies multi-head attention mechanism, which takes edge information in the graph structure into consideration. Compared with improving GCN (Hong et al., 2020), we do not mutually exclusive allocate separate subspaces for each relation type, which is more effective for extracting overlapping relations. The difference is that we also use the tightly connected GCN in our work, which enables our model to extract deeper graph structure information for learning the relations between entity pairs.

Experimental results demonstrates that our model's comprehensive performance F1 value is higher than that of all baseline models, positive to ImprovingGCN 6.6% on NYT and positive to AntNRE (Sun et al., 2019) 8.3% on WebNLG. AntNRE removes irrelevant edges in bipartite graphs by relational binary classification task, and their performance depends on binary classification task. For “Precision” and “Recall,” on the NYT data set, ours' precision is only 1.9% lower than the highest ImprovingGCN, but ours' recall is 12% higher than it. Compared with the other seven baseline models, ours' precision and recall are superior. Similar trend could be seen on the WebNLG dataset. On the other hand, our model can fully recognize the boundary of the entity while CopyRe cannot copy the complete entity. Therefore, the F1 score of CopyRe on the two datasets NYT and WebNLG is 20.7 and 29.2% lower than ours. AntNRE constructs an entity-relation bipartite graph, but the performance will be affected by the binary classification task of the nodes in the bipartite graph. Although ImprovingGCN and AntNRE consider the dependency information of words and the interaction of triplets, they ignore the interaction between words in different relational spaces. On the contrary, our proposed relation adaptive mechanism can capture the hidden connections of words in different relational spaces, and then establish a chain of reasoning between triplets. Therefore, it is proved that our work is meaningful and the comprehensive performance of our proposed model is superior.

### Ablation Study

In order to verify the validity of each component in the model, as NYT data sets overlap far more than WebNLG data sets, we conducted ablation experiments on NYT data sets. The impact of different components on model performance was compared by removing one component at a time. The experimental results can be obtained from Table 4.

**Table 4:** Ablation tests on the NYT dataset.

**Model**	**F1 (%)**
ALL	79.4
- char embedding	78.1
- context embedding	77.8
- BiGCN	77.6
- Multi-head Attention	76.9
- DCGCN	78.2

As can be seen from Table 4, F1 value of the model decreases by 1.3% when character-based word features are not added, and by 1.6% when context embedding is not added. The results indicate that the multi-granularity embedding can provide more semantic features of words and improve the performance of the model to some extent. “Bi-GCN” indicates that the model removes the GCN component of the encoding layer, leaving only Bi-LSTM. The experimental results show that the stacked LSTM-GCN encoding layer can extract richer word features for named entity recognition and relation extraction than the single LSTM. The results show that the performance decreases by 1.5% after removing the attention mechanism, which proves the effectiveness of the relational adaptive layer in detecting overlapping relations. Attention weights can provide more effective edge features to make the model learn the interaction information of entity relations better, and the non-mutually exclusive subspace of each relation also makes it easier for the model to learn the overlapping relation between entities. Where “-DCGCN” means to use the same regular Bi-GCN as before instead of densely connected GCN. The results show that DCGCN can aggregate nodes more effectively and provide deeper graph structure information.

### Comparison Results for Different Numbers of GCN Layers

In order to select the best match of GCN layers in the two stages, we set different numbers of GCN layers in first-phase and second-phase to carry out comparison experiment on NYT dataset. The experimental results are shown in Table 5.

**Table 5:** F1 score for different numbers of GCN layer.

**Phase**	**# Numbers of layer**	**F1 (%)**
1st-GCN	1	76.8
	2	**77.6**
	3	77.3
2nd-DCGCN	2	79.1
	3	**79.4**
	4	79.3
3rd-DCGCN	3	79.3

*Bold marks the optimal setting*.

When conducting experiments on the number of layers in the first-phase GCN, our model does not use the second-stage DCGCN, but only retains first-phase GCN. This experimental data also shows the effectiveness of setting two-stage GCN of our model. The DCGCN of second-phase can extract more information to improve the performance of the model. When DCGCN in the second and third phase conducts experiments with different number of layers, the previous GCN is set to the optimal value obtained from the experiment, such as layers in first-phase and second-phase set to two and three.

As shown in Table 5, we also tried to add the third-phase DCGCN again for relation extraction, but the result declined instead. This indicates that GCNs of more phase cannot achieve better results, and the information of the graph structure will become smooth after multiple GCNs. The setting of layer (2,3) is the most suitable match for our model.

### Comparison Results for Overlapping Triples

**Figure 3:**
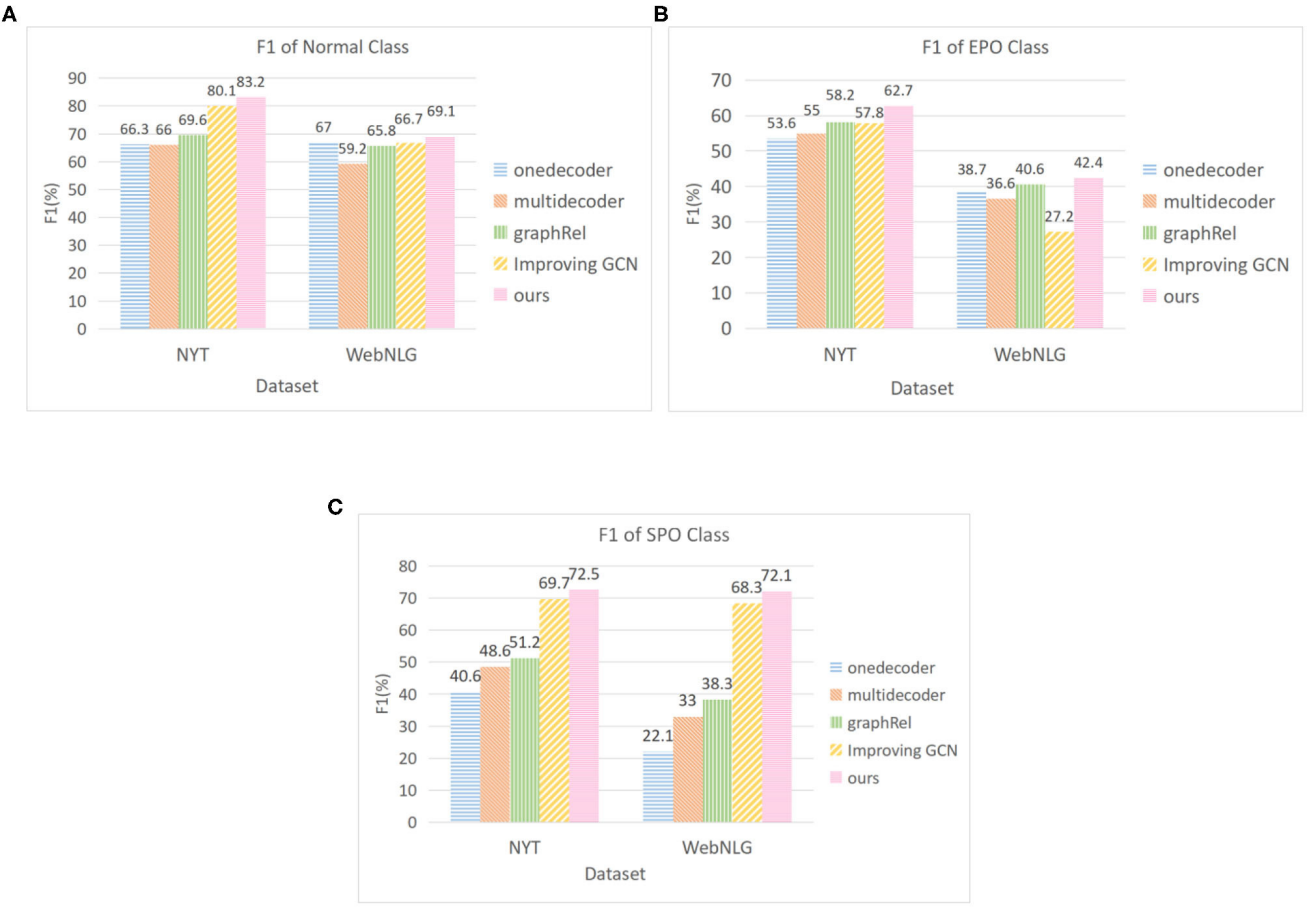
F1 score for different class of overlapping triples. **(A)** F1 of normal triple **(B)** F1 of EntityPairOverlap **(C)** F1 of SingleEntityOverlap.

Figure 3 shows the performance comparison of the model in this paper for different types of overlapping triples on two public datasets. Referring to GraphRel's work (Fu et al., 2019), we compare it with two encoder models (Zeng et al., 2018) and two similar methods (Fu et al., 2019; Hong et al., 2020). The experimental results indicate that the detection performance of our model is better than that of all baseline models for the three types of triples. Especially compared with GraphRel, the F1 value of NYT and WebNLG for SPO increased by 21.3 and 33.8%, respectively. Our model uses multi-stacked GCN to better extract the interaction information between entities and relations, and the introduction of multi-head attention mechanism is more targeted to detect overlapping triples.

**Figure 4:**
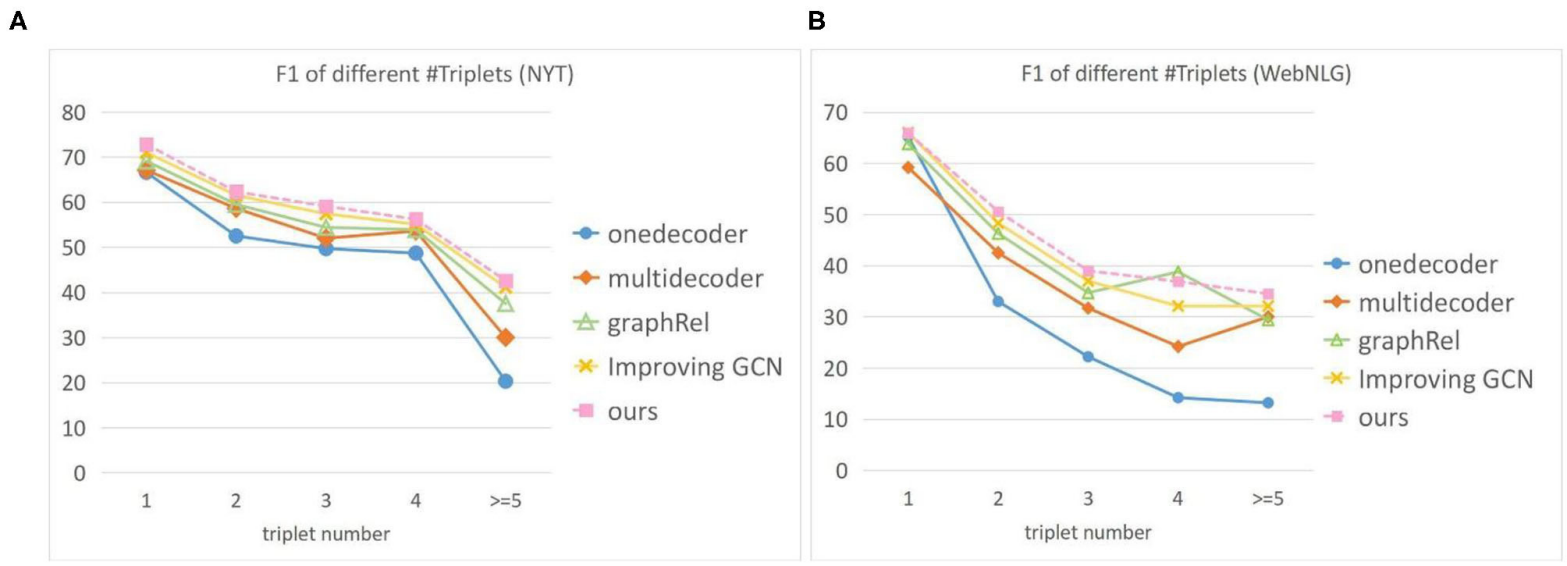
F1 score for different number of triples on two datasets. The X-axis represents the number of triples in a sentence. **(A)** F1 of different triples on the NYT **(B)** F1 of different triples on the WebNLG.

We also conducted a comparison experiment on two public datasets with different number of triples in a sentence. The results are shown in the Figure 4. Where the *x*-axis represents the number of triples in a sentence. In most cases, our model is superior to other baseline models. With the increase of the number of triples in the sentence, the performance of each model began to decline, and the decline of our model was smaller. On the WebNLG dataset, although F1 of GraphRel is slightly higher than our model when the sentence contains four triples, its F1 value drops sharply when the sentence contains three and five triples. This indicates that the overall performance of our model is more stable. As the number of triples in the sentence increases, the graph will be built with more nodes. That means our model can extract the graph structure information at a deeper level than the other model, which is more suitable for extracting richer information from larger graphs.

## Conclusion

We propose a new joint entity and relation extraction model based on densely connected graph convolutional network (DCGCN). We introduce a multi-head attention mechanism to assign independent attention weights to different relations that are not mutually exclusive, and adaptively extract multiple relation types between overlapping entity pairs. In order to further strengthen the interaction between entities and relations, a stacked DCGCN is added to the model, and the features of adjacent nodes and weighted edge information are used to extract more hierarchical graph structure information. We evaluated our approach on two public datasets. The results show that we can achieve the most advanced performance compared to current mainstream methods. In the future work, we hope to make more effective use of the rich semantic information in the pre-training model to improve model's performance, such as inputting the trained attention weight in the pre-training model into our proposed model.

## Data Availability Statement

Publicly available datasets were analyzed in this study. This data can be found here: https://drive.google.com/file/d/1kAVwR051gjfKn3p6oKc7CzNT9g2Cjy6N/view; https://drive.google.com/file/d/1zISxYa-8ROe2Zv8iRc82jY9QsQrfY1Vj/view.

## Author Contributions

GD and JM designed the model and completed part of the experiment. TH and WL completed part of the experiment, and DH completed the article writing. All authors contributed to the article and approved the submitted version.

## Conflict of Interest

The authors declare that the research was conducted in the absence of any commercial or financial relationships that could be construed as a potential conflict of interest.
